# Opioid Overdose Education for Individuals Prescribed Opioids for Pain Management: Randomized Comparison of Two Computer-Based Interventions

**DOI:** 10.3389/fpsyt.2018.00034

**Published:** 2018-02-12

**Authors:** Andrew S. Huhn, Albert Perez Garcia-Romeu, Kelly E. Dunn

**Affiliations:** ^1^Behavioral Pharmacology Research Unit, Johns Hopkins School of Medicine, Baltimore, MD, United States

**Keywords:** opioids, overdose, naloxone, overdose prevention, overdose education

## Abstract

**Background:**

Opioid overdose (OD) rates in the United States have reached unprecedented levels. Current OD prevention strategies largely consist of distribution of naloxone and in-person trainings, which face obstacles to expedient, widespread dissemination. Web-based interventions have increased opioid-OD response knowledge in patients with opioid-use disorders; however, these interventions have not been tested in the larger population of individuals that are prescribed opioid analgesics. This study assessed a web-based intervention providing education across three knowledge domains: opioid effects, opioid-OD symptoms, and opioid-OD response.

**Methods:**

Participants (*N* = 197) were adults recruited on Amazon Mechanical Turk from May to June 2017, who were prescribed an opioid medication for pain. Participants were randomly assigned to a Presentation (*n* = 97) intervention communicating relevant facts in each knowledge domain, or a Presentation + Mastery (*n* = 100) intervention including the same facts but requiring that participants respond correctly to ≥80% of embedded questions in each module before advancing. Participants completed the Brief Opioid Overdose Knowledge (BOOK) measure before and after the interventions, and provided feedback on acceptability.

**Results:**

Both versions of the intervention resulted in significant pre to postintervention increases in BOOK scores across all knowledge domains (*p* < 0.001), with no significant knowledge differences between groups. The Presentation intervention took significantly less time to complete (*p* < 0.001) and was completed by significantly more participants than the Presentation + Mastery intervention (*p* < 0.001). Most participants rated both interventions as highly acceptable.

**Conclusion:**

Results replicate a previous study (1) and suggest the web-based Presentation intervention may be a convenient, cost-effective method for disseminating crucial public health information for preventing opioid OD.

## Introduction

The opioid epidemic continues to claim an increasing number of American lives each year (2). Drug poisonings, which are largely driven by opioid exposures, are currently the leading cause of accidental death among adults aged 25–64 (3), with approximately 91 opioid overdoses (ODs) occurring each day in the United States (4). Opioid-OD interventions focus largely on the provision of naloxone (Narcan), a fast-acting opioid antagonist that is FDA-approved for opioid-OD reversal, and training of non-medical bystanders in methods to prevent, recognize, and respond to an opioid-related OD. While communities that implement OD intervention trainings have reported reduced OD deaths (5–7), the ability to scale up these approaches is somewhat limited by the manner in which they are administered. Specifically, most interventions are conducted using in-person trainings in small-group settings, which requires specially trained personnel to deliver the content, as well as adequate space and time to conduct the trainings. However, a public health response that is proportional to the opioid epidemic requires smart and scalable approaches to deliver educational content focused on opioid-OD prevention.

Many OD educational strategies have targeted individuals with opioid-use disorder (1, 8); however, there is a much larger population of individuals who are prescribed opioids for pain management who never develop opioid-use disorder (9). Educational strategies and informed consent for opioid therapy are especially important for these patients (10) to mitigate the risk of opioid OD (11, 12). Co-occurring chronic pain and opioid misuse increases the risk of opioid OD (13), and individuals using prescription opioids for pain may have lower baseline knowledge concerning risks such as accidental OD (14). Individuals with chronic pain are also more likely to be engaged in ongoing medical care, and thus a standardized intervention could be used to reach this population in a way that is consistent and effective. Community-based programs have been effective in distributing naloxone (15). Nevertheless, these programs have rarely targeted chronic pain patients (16); instead, they have largely focused on individuals that have recently overdosed (17), individuals with opioid-use disorder, or laypersons that might be in position to witness an OD (18). Pairing a computerized intervention with opioid medication or naloxone distribution could have a major impact on reducing the number of OD deaths attributable to prescription opioids.

We recently evaluated three different modalities for increasing knowledge domains related to opioid OD. These knowledge domains targeted general knowledge about opioids, identifying opioid ODs and responding to opioid ODs among 76 patients in treatment for opioid-use disorder at a hospital-based, outpatient detoxification unit in Baltimore, MD, USA (1). The first intervention (Presentation) was a web-based presentation-only method, wherein participants reviewed information related to the aforementioned three domains. The second (Presentation + Mastery) presented participants with the same Presentation intervention but required them to correctly answer questions embedded within the slides to advance through the program. Finally, the third intervention (Pamphlet) was a printed pamphlet that included the same information as the web-based presentations and served as an ecological control condition. The primary outcome was measured *via* a 52-item OD knowledge test before and after the intervention, and at a 1- and 3-month follow-up. Participants rated all three interventions as acceptable, with the web-based groups rating their interventions as slightly easier to understand and less confusing than the pamphlet group. All three methods also led to significant pre–post knowledge increases on the three domains tested. The most striking difference was observed on opioid-OD response knowledge, and although there were no pretest-to-posttest group differences, there was a main effect such that the mean percent of correct responses increased from 41.8 to 73.8% between the pretest and posttest, respectively. Knowledge retention at the 1- and 3-month follow-ups was slightly higher in participants who underwent the web-based vs. pamphlet condition, but did not vary between the two web-based conditions; the interpretation of these results is limited as 43% of the sample was lost to follow-up (1). Another study on computerized delivery of overdose prevention education (Overdose Risk InfOrmatioN, ORION) in the United Kingdom, Denmark, Italy, and Germany did not find differences between pretest-to-posttest scores in treatment-seeking, opioid-dependent individuals (19), but did find an inverse correlation between risky behaviors and perceived self-efficacy to reduce overdose risk. The interventions utilized between our study (1) and the ORION study (19) were different, as our study emphasized identifying and responding to OD rather than modifying personal risky behaviors. Web-based interventions for treatment-seeking adults might be more effective at training individuals to respond to an opioid OD as opposed to behavior modification.

The results from our study in treatment-seeking adults were promising and indicated that a web-based method for delivering information may be useful for increasing knowledge about opioid OD. Web-based delivery of information has several potential advantages relevant to the opioid epidemic, including that information is always relayed in a uniform manner, does not require specialized staff training for administration, and can be accessed from any location with internet access, suggesting that it may be more amenable to scaling than in-person trainings. However, that study had some notable limitations. First, participants entered the study with relatively high levels of baseline knowledge of opioids and opioid OD, which may have resulted in a ceiling effect against which additional gains in knowledge in those domains was not possible (1). Research indicates that patients with opioid-use disorder may have greater opioid-related knowledge relative to patients receiving opioids for the treatment of chronic pain (13, 14), suggesting that a pain management group may be an important target for educational interventions. Second, the Presentation + Mastery intervention in the prior study did not provide corrective feedback when participants answered questions incorrectly, which may have undermined educational effects since corrective feedback is known to be a valuable component of mastery interventions (20, 21). Finally, at the time of the previous study, there were no standardized measures that were appropriate for assessing changes in OD knowledge.

The current study sought to expand upon the previous study by comparing pre–post knowledge gains following randomization to the two web-based interventions (Presentation, Presentation + Mastery) among participants who report taking an opioid medication for pain management but do not necessarily have opioid-use disorder. The web-based versions were selected for comparison based upon their more favorable ratings in the previous study and their potential value for providing standardized and remote access to information regarding opioid ODs. Participants in this study who were assigned to the Presentation + Mastery condition were also able to receive corrective feedback about their performance on embedded questions. Finally, in the current study, changes in pretest-to-posttest knowledge were evaluated using the Brief Opioid Overdose Knowledge (BOOK) measure (14), a 12-item self-report OD knowledge measure validated in both opioid-use disorder and chronic-pain patients. Hypotheses for the present study were that both interventions would increase pre–post knowledge, that the Presentation + Mastery condition would result in greater increases overall, and that both methods would be considered acceptable by a pain management population.

## Materials and Methods

### Participants

Participants (*N* = 202) were recruited between May 2017 and June 2017 from the crowdsourcing website Amazon Mechanical Turk (MTurk). MTurk “workers” responded to a Human Intelligence Task (HIT) advertisement for a survey on health behaviors (22, 23), which was only open to workers with a ≥90% approval rate from completion of previous HITs and who resided in the United States. The specific nature of the intervention was blinded to prevent falsification of responses. All participants completed a brief introductory survey to assess their eligibility for the study, and only participants who reported being aged 18 or older and currently prescribed an opioid medication (with several examples given) for pain were advanced to the intervention. A total of *N* = 1,469 participants were screened for eligibility and *N* = 202 (13.8% of the screened sample) were enrolled and randomly assigned to an intervention condition. Participants who met eligibility criteria were presented with a screen that described the study in detail and required them to select “Yes” to proceed into the study or “No” to end their participation. For quality control purposes, participants were also asked whether their data should be included in study analyses. Five participants answered “no” to an item asking if they had answered the majority of questions accurately so were removed from analyses, leaving a final sample size of *N* = 197. Participants were compensated $0.10 for completing the eligibility survey and $5.00 for completing the intervention study. Since no protected health information was collected, the Johns Hopkins University Internal Review Board categorized this study as exempt from human subject’s research.

### Measures

#### Baseline Characteristics

Participants completed a brief demographics measure. After completing the intervention, participants were asked whether they had ever experienced an opioid-related OD, which was described to them as “An overdose occurs when you take too high a dose of opioids, and it is not always fatal. Please answer these questions even if you are NOT SURE whether you ever overdosed on these medications, but know that you had a bad or scary experience from taking them.” Participants were also asked whether they had ever previously heard of naloxone (Narcan), had received a prior prescription for naloxone, or had been trained to administer naloxone or CPR.

#### Brief Opioid Overdose Knowledge (BOOK) Questionnaire

The primary outcome measure in this study was the BOOK questionnaire, which was administered prior to the intervention and immediately after completion of the intervention (14). The BOOK asks 12 questions from three domains: general opioid knowledge (four items), opioid-OD knowledge (four items), and opioid-OD response knowledge (four items). Results are rated as True, False, or I Don’t Know and are summed to create three subscales (score range 0–4 for each subscale) and a Total score (range 0–12) rating. Items answered as “I don’t know” were coded as incorrect when summing the Total and subscale scores, and changes in the number of items answered “I don’t know” were also compared across groups as another measure of learning.

#### Acceptance Questionnaire

Upon completing the intervention, participants were asked to rate their acceptance of the intervention. The following items were rated on a scale from 1 (strongly agree) to 5 (strongly disagree): “The education intervention …”: “was helpful,” “taught me information I didn’t know before,” “was easy to understand,” “was fun,” “was too long,” “was interesting,” and “was confusing.” An additional three items were also rated on a similar 1–5 scale: “I would recommend this intervention to someone else,” “I believe that more people should receive this educational intervention,” and “I do not think this educational intervention was useful.” Finally, participants were asked whether they believed this intervention would help prevent them from overdosing in the future (yes/no), whether it would change the way they would help other people who are overdosing (yes/no), how important they believe it is to learn how to prevent, recognize, and respond to an OD (very, somewhat, or not important), and whether they would recommend the intervention to a family member or friend (yes/no).

### Interventions

Both the interventions used here were identical to those used previously (1) with the exception that the Presentation + Mastery intervention now provided corrective feedback whenever a participant answered a question incorrectly. Both interventions were hosted through the online survey manager Qualtrics (Provo, UT, USA). Each presented three slides to introduce the participant to the computerized system before presenting 25 educational content slides that combined text, pictures, and/or videos in the domains of general opioid knowledge, opioid-OD knowledge, and opioid-OD response knowledge. No restrictions were placed on the length of time the participants took to complete either intervention and time for completion was calculated automatically within Qualtrics.

The Presentation Intervention presented information for viewing and required no additional interaction with the program; participants were able to move through slides at their own pace. The Presentation + Mastery intervention required participants to achieve ≥80% accuracy on 114 questions that were embedded throughout the intervention and focused on opioid knowledge (52 questions), opioid-OD knowledge (46 questions), and opioid-OD response knowledge (16 questions). Embedded questions were different from BOOK questions to prevent improvements in BOOK scores from being related to corrective feedback. Failure to meet the ≥80% threshold prompted the module to start over, which happened a maximum of three times before the participant was automatically advanced to the next module.

### Statistical Analysis

The study hypothesized that both the Presentation and the Presentation + Mastery interventions would increase knowledge on the post-BOOK measure relative to a pretest, and that the Presentation + Mastery intervention would lead to greater gains overall. It was also hypothesized that both interventions would be highly rated on acceptability.

Baseline demographic variables, time to complete the intervention (minutes), and prior OD and naloxone experiences were compared across groups using independent groups *t*-tests and chi-square analyses for continuous and dichotomous variables, respectively. A Bonferroni-correction was used for the acceptability items that were rated on the same scale. Correcting for those comparisons, the Bonferroni-corrected statistical significance threshold for those items was *p* = 0.008. A Mann–Whitney *U* test was used to compare median household income. Main effects and group interactions between pretest and posttest scores were examined using repeated measures ANOVAs for the three subscales, the number of items rated as “I don’t know,” and the BOOK total score. These analyses controlled for significant between-group differences in naloxone knowledge and/or training [i.e., *have you ever heard of naloxone* and *have you been trained to administer naloxone* (Table 1)]. Attrition, defined as failure to complete the intervention, was compared across groups as a proxy measure of acceptability using chi-square analyses. Individuals who discontinued participation prior to finishing the intervention did not receive a posttest or acceptability survey to complete and therefore could not be included in analyses. To maintain consistency with the previous evaluation of these interventions, additional acceptability questions were dichotomized such that 1 = agree or strongly agree and 0 = neutral, disagree, and strongly disagree, and results were compared across groups using chi-square analyses. Alpha levels for significant findings were set at *p* < 0.05 and analyses were conducted using SPSS version 24.0.

**Table 1:** Participant characteristics.

	Total sample (*N* = 197)	Presentation (*n* = 97)	Presentation + mastery (*n* = 100)	χ^2^/U/*t*-value (*p*-value)
**Demographics**				
Age M (SD)	33.2 (11.4)	32.7 (11.1)	33.7 (11.6)	0.63 (0.53)
Female (%)	45.2	39.2	51.0	2.78 (0.10)
Median household income	$52,500	$52,500	$38,500	4,544 (0.45)
**Opioid characteristics (%)**				
Misused prescription opioid in previous year	39.1	34.0	44.0	2.01 (0.15)
Overdosed on opioids ≥1	19.5	24.7	13.2	3.57 (0.06)
Heard of naloxone	68.5	60.9	77.6	**5.42 (0.02)**
Received naloxone prescription	5.4	6.5	3.9	0.55 (0.46)
Trained to administer naloxone	7.1	10.9	2.6	**4.26 (0.04)**
Trained to administer CPR	64.9	66.3	63.2	0.18 (0.67)

*Comparisons based upon independent group t-tests for continuous and chi-squares for dichotomous variables. Participants were recruited via Amazon Mechanical Turk from May to June 2017*.

significant differences in bold

## Results

### Participants

Participants were randomly assigned into the Presentation (*n* = 97) and Presentation + Mastery (*n* = 100) groups. Demographics and results of between-group comparisons are presented in Table 1. Participants in the study were 54.8% female and had a median household income of $52,500. Participants had been prescribed opioids for pain management for a median of 4 months (range: less than a week to more than 10 years). Approximately 19.5% of participants reported overdosing from opioids at least once in their lifetime. A total of 68.5% of participants had heard of naloxone, 5.4% had received a prescription for naloxone, 7.1% had been trained to administer naloxone, and 64.9% had received training in CPR. Of the participants who completed the intervention (*n* = 168), those in the Presentation group spent a mean (SD) of 21.5 (12.3) min completing the intervention, which was significantly shorter than those assigned to the Presentation + Mastery group, who required a mean (SD) of 34.7 (14.0) min to complete the intervention [*t*(166) = 6.47, *p* < 0.001].

### BOOK Test Outcomes

**Figure 1:**
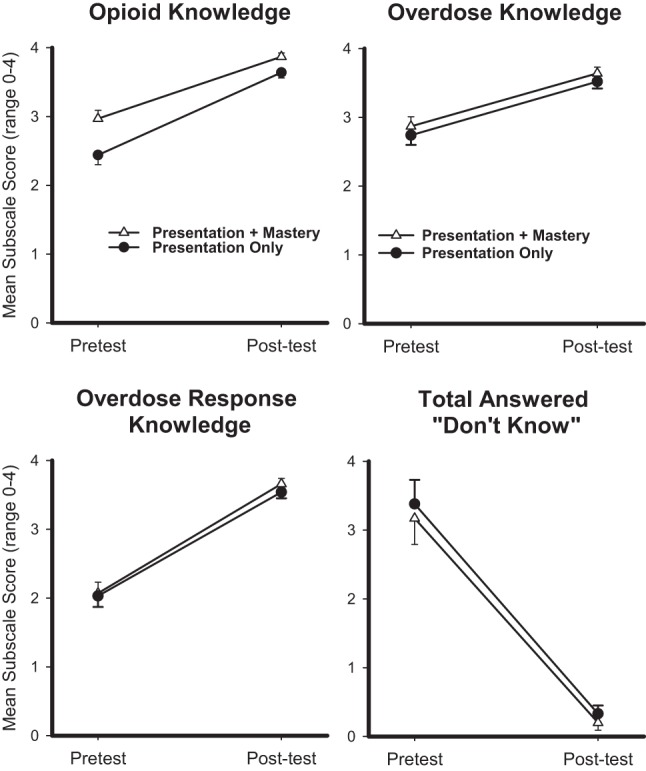
Mean pretest and posttest scores are displayed for opioid knowledge (top left), overdose knowledge (top right), overdose response knowledge (bottom left), and number of items rated “I Don’t Know” (bottom right) from the Brief Opioid Overdose Intervention (BOOK) for the Presentation (circle) and Presentation + Mastery (triangle) groups. *x*-axes represent pretest and posttest time points, *y*-axes represent mean response (range 0–4 for each item), and error bars represent standard error of the mean. Repeated measures ANOVAs revealed that main effects were significant for each outcome at *p* < 0.001. No interaction effects were observed.

Changes in pre- and post-scores from the BOOK were examined to assess the degree to which opioid knowledge, OD knowledge, and OD response knowledge changed as a result of the intervention, as well as whether any significant group interactions existed. Results showed significant gains in all three domains following both interventions. Main effects between pretest and posttest scores were evident on the BOOK total score [*F*(1,164) = 129.1, *p* < 0.001], which increased significantly, as well as the number of responses that were answered as “I don’t know” [*F*(1,164) = 104.5, *p* < 0.001], which decreased significantly between assessments (Figure 1). Main effects between the pre- and post-administrations were also evident on each of the BOOK subscales, with mean pre-to-post increases in opioid knowledge [*F*(1,164) = 80.4, *p* < 0.001], opioid-OD knowledge [*F*(1,164) = 32.0, *p* < 0.001], and opioid-OD response knowledge scores [*F*(1,164) = 121.6, *p* < 0.001] (Figure 1). There were no interaction effects or between-group differences in pre-to-post test scores.

### Intervention Acceptability

Between-group comparisons of acceptability ratings are presented in Table 2. Overall, both groups rated their respective interventions as highly acceptable, with 88.1% of all participants stating the intervention would help prevent them from overdosing, 94% stating it would change the way they would respond to other people overdosing, and 92.9% stating they would recommend it to a family member or friend. There were significant differences in intervention completion, with participants in the Presentation group (94.8%, *n* = 92) being significantly more likely than participants assigned to the Presentation + Mastery group (75%, *n* = 75) to complete the intervention [χ^2^(1) = 15.02, *p* < 0.001]. There were few differences in acceptability ratings. Almost twice as many participants in Presentation + Mastery endorsed that the intervention took too long compared with participants in the Presentation group [29.3 vs. 15.1%, respectively, χ^2^(1) = 5.03, *p* = 0.03]. This finding was not significant at the Bonferroni-corrected threshold of *p* = 0.008. No additional between-group differences were identified.

**Table 2:** Intervention acceptability.

Questions on usefulness of the overdose prevention program	All Participants (% yes) (*N* = 168)	Presentation only (% yes) (*n* = 93)	Presentation + Mastery (% yes) (*n* = 75)	χ^2^(1) (*p*-value)
The educational intervention was helpful	91.0	87.1	96.0	4.05 (0.04)
The educational intervention taught me information that I did not know before	86.9	83.8	90.1	1.69 (0.19)
The educational intervention was easy to understand	91.1	88.2	94.7	2.15 (0.14)
The educational intervention was fun	56.5	57.0	56.0	0.02 (0.90)
The educational intervention took too long	21.4	15.1	29.3	5.03 (0.03)
The educational intervention was interesting	83.9	80.6	88.0	1.67 (0.20)
The would recommend this educational intervention to someone else	84.5	82.8	86.7	0.05 (0.49)
I believe that more people should receive this educational intervention	89.3	86.0	93.3	2.32 (0.13)
I do NOT think the educational intervention was useful	4.8	6.5	2.7	1.31 (0.25)
The educational intervention was confusing	6.5	5.4	8.0	0.47 (0.49)
Intervention will help prevent you from overdosing	88.1	89.2	86.7	0.26 (0.60)
Intervention will change the way you help other people who are overdosing	94.0	94.6	93.3	0.12 (0.73)
I would recommend this intervention to a family member or friend	92.9	91.4	94.7	0.67 (0.42)

*Values represent the % of participants that endorsed “agree” or “strongly agree” to the statements listed, unless otherwise specified. Data collected only from individuals who completed the full intervention (*N* = 168; 85.3% of the evaluated sample). Bonferroni correction was used for the first six questions as they were presented as part of the same question: significance threshold = 0.008. Participants were recruited *via* Amazon Mechanical Turk from May to June 2017*.

## Discussion

It is essential that individuals exposed to opioids, whether through valid prescriptions or illicit means, have fundamental knowledge of opioid use, opioid-OD risk behaviors, and opioid-OD response behaviors to help prevent fatal ODs. Patients who are being prescribed an opioid for the treatment of pain management have been identified as a high-risk group due to their continued access to opioids and lower relative knowledge of risks compared with illicit users (11, 13, 14). This study refined and evaluated an educational intervention that was previously shown to be effective in increasing OD knowledge and prevention strategies in detoxified opioid-use disorder patients (1) and expanded it to patients receiving opioids for pain management. Despite being collected from a different population, the results of this study replicated several aspects of the previous study, including pre-to-post knowledge gains, high levels of acceptability, and time required to complete the Presentation intervention (16.3 min in the previous study vs. 21.5 min in the current study). These data support large-scale evaluation and subsequent utilization of the Presentation version of the educational intervention described in this study.

In the current study, main effects in learning were evident for both the Presentation-only and Presentation + Mastery groups as measured by the BOOK total score and subscales (Figure 1). Specifically, both groups had significant gains in knowledge following intervention completion, though no between-group differences were evident. This is somewhat unexpected since the Presentation + Mastery group was provided with corrective feedback when answering questions incorrectly, which has been empirically shown to promote additional knowledge gains. It is possible that the short overall duration (i.e., <1 h) between pretest and posttest in this study may have attenuated the possibility of observing any educational differences between interventions, and that such effects may be more evident in longer term follow-ups of knowledge retention. This remains to be empirically tested, but represents a methodological limitation of the current study to be addressed in future research. Nevertheless, these results suggest that participants who completed the educational intervention were able to gain essential knowledge relating to opioid OD regardless of the form of intervention to which they were assigned. Participants from both groups also overwhelmingly rated the interventions as acceptable, with more than 90% stating they would recommend it to family members or friends (Table 2). It is notable that participants who were assigned to the Presentation + Mastery group were less likely to complete the intervention than those assigned to a simple Presentation method (Table 2). This is likely related to differences in the length of time required to complete the intervention, as well as potential frustration related to answering embedded questions incorrectly. Overall, acceptability findings suggest high feasibility of a web-based intervention like the one described in this study that could be delivered in an office-based, hospital, school, or home setting. In addition, physicians who treat chronic pain might make opioid prescriptions contingent on a computerized intervention in order to enhance patient knowledge of risk behaviors, OD prevention, and OD response.

This study has some limitations. First, it did not collect follow-up data so could not evaluate potential differences in knowledge retention over time. It also did not have any mechanism for assessing whether the knowledge gains translated to changes in behavior. For instance, in addition to knowledge it is important to determine that participants are able to accurately administer a sternal rub or naloxone following intervention completion. Future studies are needed to examine knowledge retention and whether computerized interventions result in demonstrable shifts in OD rescue behaviors. In addition, there was no non-web-based control group included in this intervention, though this is somewhat mitigated by previous studies that have established brief interventions as effective (8) and the fact that this study was explicitly evaluating which of these two web-based interventions was best suited for pain management patients. Finally, no power analysis was conducted and the online nature of participant recruitment and intervention delivery precluded objective verification of eligibility criteria.

## Conclusion

Patients receiving opioids for pain management may be at increased risk for opioid OD, and results from the current study suggest that the Presentation version of our educational intervention may have value for dissemination to mitigate the risk of fatal OD. In the current study, the Presentation intervention resulted in significant pre-to-post knowledge gains and was rated as highly acceptable. Significantly more participants in this study were also willing to complete the Presentation form of the intervention, relative to the Presentation + Mastery group. In addition, the results from this study replicate the results of a previous study in patients being treated for opioid-use disorder, which reported greater post-intervention knowledge retention among participants who received either of the current web-based interventions relative to a pamphlet ecological control. That the Presentation intervention was able to produce significant gains in knowledge following a 16–22 min session that was completed remotely (which is the manner in which it would be completed in a non-research setting) suggests that this intervention may be an efficient and scalable method for providing individuals who are exposed to opioids through either licit or illicit means with valuable information related to opioid-OD prevention. Additional research regarding longer term knowledge retention and demonstration of behavioral skill building following exposure is warranted.

## Ethics Statement

This study was exempt from human subject’s research requirements by the Johns Hopkins Internal Review Board because recruitment was done online and no protected health information was collected. The study was acknowledged under a larger umbrella protocol (IRB 00054890) but does not qualify as human subject’s research. The population described in this manuscript is not recognized as a vulnerable population.

## Author Contributions

All authors contributed to the research and writing of the work described in this manuscript. AH was responsible for data analysis.

## Conflict of Interest Statement

The authors declare that the research was conducted in the absence of any commercial or financial relationships that could be construed as a potential conflict of interest.
